# Factors predicting dysphagia after anterior cervical surgery: A multicenter retrospective study for 2 years of follow-up

**DOI:** 10.1097/MD.0000000000007916

**Published:** 2017-08-25

**Authors:** Tao Wang, Lei Ma, Da-Long Yang, Hui Wang, Zhi-Long Bai, Li-Jun Zhang, Wen-Yuan Ding

**Affiliations:** a Department of Orthopaedics, The Ninth People Hospital of Wuxi, Wuxi; b Department of Orthopaedics, The First Hospital of HanDan, HanDan; c Department of Orthopaedics, The First Hospital of Shijiazhuang, Shijiazhuang; d Department of Spinal Surgery, The Third Hospital of Hebei Medical University, Shijiazhuang, China.

**Keywords:** anterior surgery, cervical, dysphagia, risk factors

## Abstract

A multicenter retrospective study.

The purpose of this study was to explore risk factors of dysphagia after anterior cervical surgery and factors affecting rehabilitation of dysphagia 2 years after surgery.

Patients who underwent anterior cervical surgery at 3 centers from January 2010 to January 2013 were included. The possible factors included 3 aspects: demographic variables—age, sex, body mass index (BMI): hypertension, diabetes, heart disease, smoking, alcohol use, diagnose (cervical spondylotic myelopathy or ossification of posterior longitudinal ligament), preoperative visual analogue scale (VAS), Oswestry Disability Index (ODI), Japanese Orthopaedic Association (JOA), surgical-related variables—surgical option (ACDF, ACCF, ACCDF, or Zero profile), operation time, blood loss, operative level, superior fusion segment, incision length, angle of C2 to C7, height of C2 to C7, cervical circumference, cervical circumference/height of C2 to C7.

The results of our study indicated that the rate of dysphagia at 0, 3, 6, 12, and 24 months after surgery was 20%, 5.4%, 2.4%, 1.1%, and 0.4%, respectively. Our results showed that age (58.8 years old), BMI (27.3 kg/m^2^), course of disease (11.6 months), operation time (103.2 min), blood loss (151.6 mL), incision length (9.1 cm), cervical circumference (46.8 cm), angle of C2 to C7 (15.3°), cervical circumference/height of C2 to C7 (4.8), preoperative VAS (7.5), and ODI (0.6) in dysphagia group were significantly higher than those (52.0, 24.6, 8.6, 88.2, 121.6, 8.6, 42.3, 12.6, 3.7, 5.6, and 0.4, respectively) in nondysphagia group; however, height of C2 to C7 (9.9 vs 11.7 cm) and preoperative JOA (8.3 vs 10.7) had opposite trend between 2 groups. We could also infer that female, smoking, diabetes, ossification of posterior longitudinal ligament, ACCDF, multilevel surgery, and superior fusion segment including C2 to C3 or C6 to C7 were the risk factors for dysphagia after surgery immediately. However, till 2 years after surgery, only 2 risk factors, smoking and diabetes, could slow rehabilitation of dysphagia.

Many factors could significantly increase rate of dysphagia after anterior cervical surgery. Operation time as a vital factor markedly increases immediate postoperative dysphagia and smoking, as the most important factor, lower recovery of dysphagia. Further study is needed to prove if these factors could influence dysphagia.

## 1 Introduction

Cervical spondylotic myelopathy (CSM) and ossification of posterior longitudinal ligament (OPLL) are common clinical degenerative diseases, seriously impacting quality of life and even causing disability for elderly population.^[1,2]^ Anterior approaches are widely applied in treatment for CSM and OPLL; however, it is difficult to avoid dysphagia.^[3,4]^ Some reported that the rate of dysphagia ranged from 2% to 60% according to different assessment.^[4–6]^ Bazaz^[7]^ reported a prospective study on the prevalence of dysphagia at 1, 2, 6, and 12 months in 249 patients who underwent anterior surgery and the incidence were 50.2%, 32.2%, 17.8%, and 12.5%, respectively.

Some authors focused on risk factors of dysphagia after anterior cervical approaches. Bazaz^[7]^ found that female patients, ≥60 years old and multiple surgeries, were risk factors for dysphagia. Olsson^[8]^ found that patients with smoking were more likely to suffer from dysphagia. Brad^[9]^ explored to compare cervical arthroplasty and anterior cervical discectomy and fusion (ACDF) with rate of dysphagia and their results showed that no-profile cervical disc arthroplasty could significantly lower rate of dysphagia compared with ACDF. But as we know, the risk factors for dysphagia after anterior cervical approaches remain debated.

This is the first multicenter retrospective study on dysphagia after anterior cervical approaches. The purpose of our study is to assess risk factors of dysphagia after anterior cervical approaches and perioperative factors predicting on rehabilitation of dysphagia.

## 2 Materials and methods

### 2.1 Subjects

The study was approved by the Institutional Review Board of the Third Hospital of Hebei Medical University, the First Hospital of HanDan, and the First Hospital of Shijiazhuang before data collection and analysis. The inclusion criteria included subjects who received anterior cervical surgery, >18 years old. The exclusion criteria include patients with cervical fracture, having history of any cervical surgery, having spinal deformities (including scoliosis, irregular endplate, sacralization, or lumbarization), and having cervical trauma or tumors. A total of 2827 patients who received anterior cervical surgery were included in our study from the Third Hospital of Hebei Medicle University, the First Hospital of HanDan, and the First Hospital of Shijiazhuang, from January 2013 to September 2015.

### 2.2 Variables

Dysphagia was assessed by Bazaz-Yoo dysphagia questionnaire.^[7]^ The patients were graded as having none, mild, moderate, or severe dysphagia based on a telephone evaluation. The patients who experienced no episodes of swallowing difficulty were graded as “none.” Patients who experienced only rare episodes of dysphagia were graded as “mild.” These patients did not feel that their dysphagia was a significant problem. Moderate dysphagia was defined as occasional swallowing difficulty with very specific foods. “Severe” dysphagia was defined as frequent difficult swallowing with the majority of foods. We considered moderate and severe dysphagia as dysphagia patients in our study. We assessed demographic variables—age, sex, body mass index (BMI): divided weight (kg) by the square of height (m), hypertension, diabetes, heart disease, smoking, alcohol use, diagnose (CSM or OPLL), preoperative visual analogue scale (VAS), Oswestry Disability Index (ODI), Japanese Orthopaedic Association (JOA), surgical-related variables—surgical option (ACDF, ACCF, ACCDF, or Zero profile), operation time, blood loss, operative level, superior fusion segment, incision length, angle of C2 to C7: the angle between upper endplate of C2 and lower endplate of C7, height of C2-C7: distance between vertebral posterior vertex of C2 and posterior vertex of C7, cervical circumference: the largest diameter of the neck, cervical circumference/height of C2 to C7.

### 2.3 Statistical analysis

The methods were carried out in accordance with the approved guidelines. Two authors identified and collected all the data of patients according to inclusion and exclusion criteria. In addition, 2 authors were responsible for data analyses. All measurement data are presented as the mean ± SD when data satisfied criteria for normality with *P* > .05. Otherwise, it should be presented as median (interquartile range, IQR). Age, BMI, preoperative VAS, ODI and JOA, operation time, blood loss, incision length, angle of C2 to C7, height of C2 to C7, cervical circumference, cervical circumference/height of C2 to C7, satisfied criteria for normality and homogeneity of variance, statistical analysis between groups were performed using independent samples *t* test. And count data, like sex, hypertension, diabetes, heart disease, smoking, alcohol use, diagnose (CSM or OPLL), surgical-related variables—surgical option (ACDF, ACCF, ACCDF or Zero profile), operative level, superior fusion segment χ^2^ test were used for data analysis. The Kolmogorov–Smirnoff test was used to verify the normal data distribution. Statistical significance levels were considered to be *P* < .05. All statistical analyses were carried out using SPSS, version 21.0 (SPSS Inc., Chicago, IL).

## 3 Results

The rate of dysphagia after anterior cervical surgery immediately was 20% (566 of 2827). Our results showed that age (58.8 ± 9.7 years old), BMI (27.3 ± 4.6 kg/m^2^), course of disease (11.6 ± 3.8 months), operation time (103.2 ± 26.5 min), blood loss (151.6 ± 23.1 mL), incision length (9.1 ± 1.2 cm), cervical circumference (46.8 ± 9.2 cm), angle of C2 to C7 (15.3 ± 3.0°), cervical circumference/height of C2 to C7 (4.8 ± 1.3), preoperative VAS (7.5 ± 1.7), and ODI (0.6 ±  0.1) in dysphagia group were significantly higher than those (52.0 ± 11.1, 24.6 ± 3.2, 8.6 ± 3.3, 88.2 ± 21.7, 121.6 ± 20.4, 8.6 ± 1.0, 42.3 ± 8.3, 12.6 ± 2.6, 3.7 ± 1.0, 5.6 ± 1.1, 0.4 ± 0.08, respectively) in nondysphagia group; however, height of C2 to C7 (9.9 ± 1.6 cm) and preoperative JOA (10.7 ± 1.8 vs 8.3 ± 1.5) had opposite trend between 2 groups. We could also infer that old subjects, female patients, individual with smoking, diabetes, OPLL, ACCDF, multilevel surgery, and superior fusion segment including C2 to C3 or C6 to C7 were the risk factors for dysphagia after surgery immediately, as shown in Table 1.

**Table 1:**
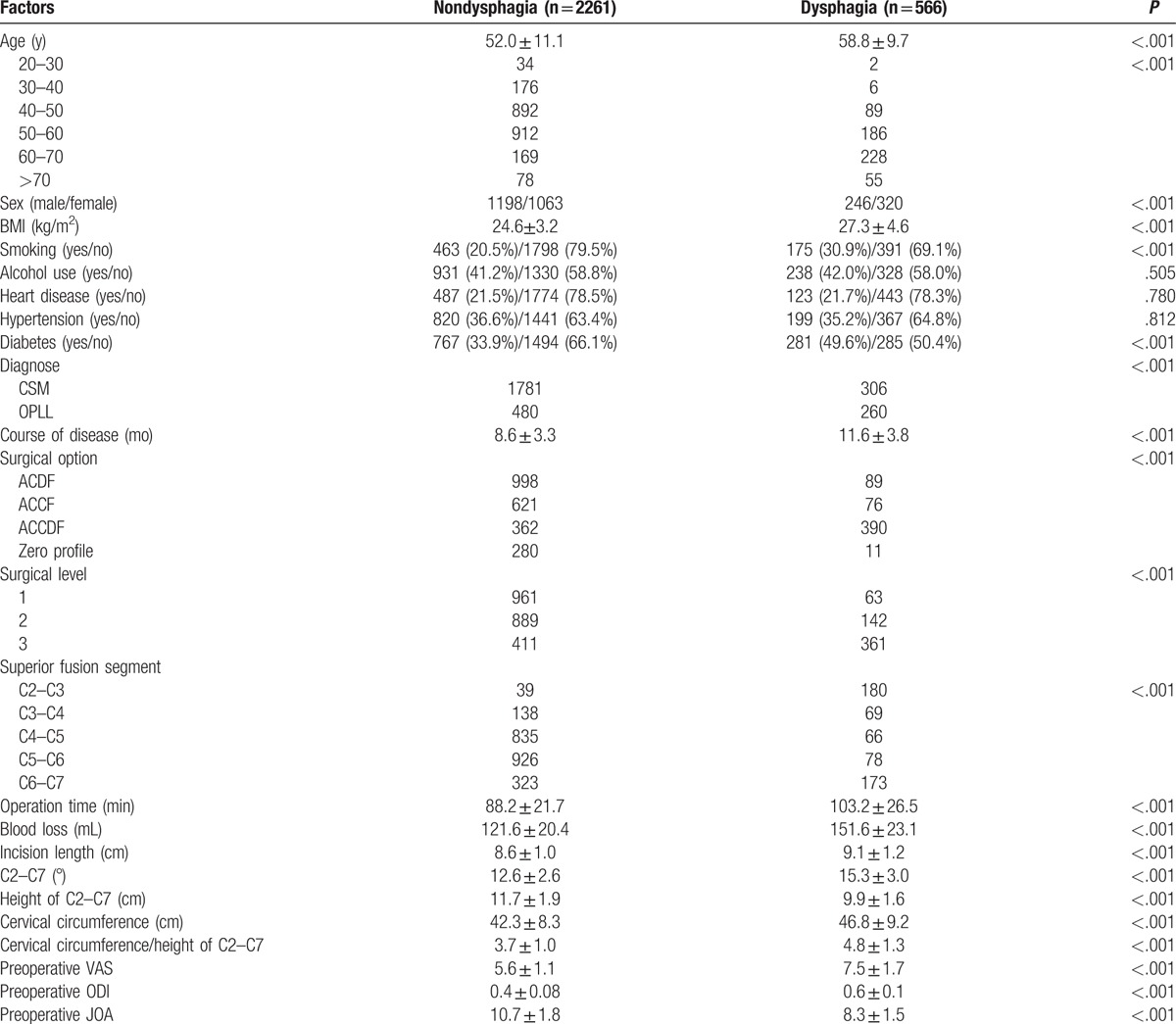
Comparison between nondysphagia group and dysphagia group after surgery immediately.

The rate of dysphagia after anterior cervical surgery 3 months after surgery was 5.4% (143 of 2827). Our results showed that age (64.4 ± 9.4 years old), operation time (111.0 ± 29.2 min), blood loss (160.5 ± 25.3 mL), preoperative VAS (7.9 ± 1.9), and ODI (0.64 ± 0.1) in dysphagia group were significantly higher than those (56.7 ± 9.0, 101.8 ± 24.5, 149.4 ± 23.8, 7.3 ± 1.6, 0.60 ± 0.1, respectively) in nondysphagia group; however, preoperative JOA (8.5 ± 1.6 vs 7.7 ± 1.3) had opposite trend between 2 groups. We could also infer that old subjects, individual with smoking, diabetes, OPLL, multilevel surgery, and superior fusion segment (C2–C3 or C6–C7) were the risk factors for dysphagia after surgery immediately, as shown in Table 2.

**Table 2:**
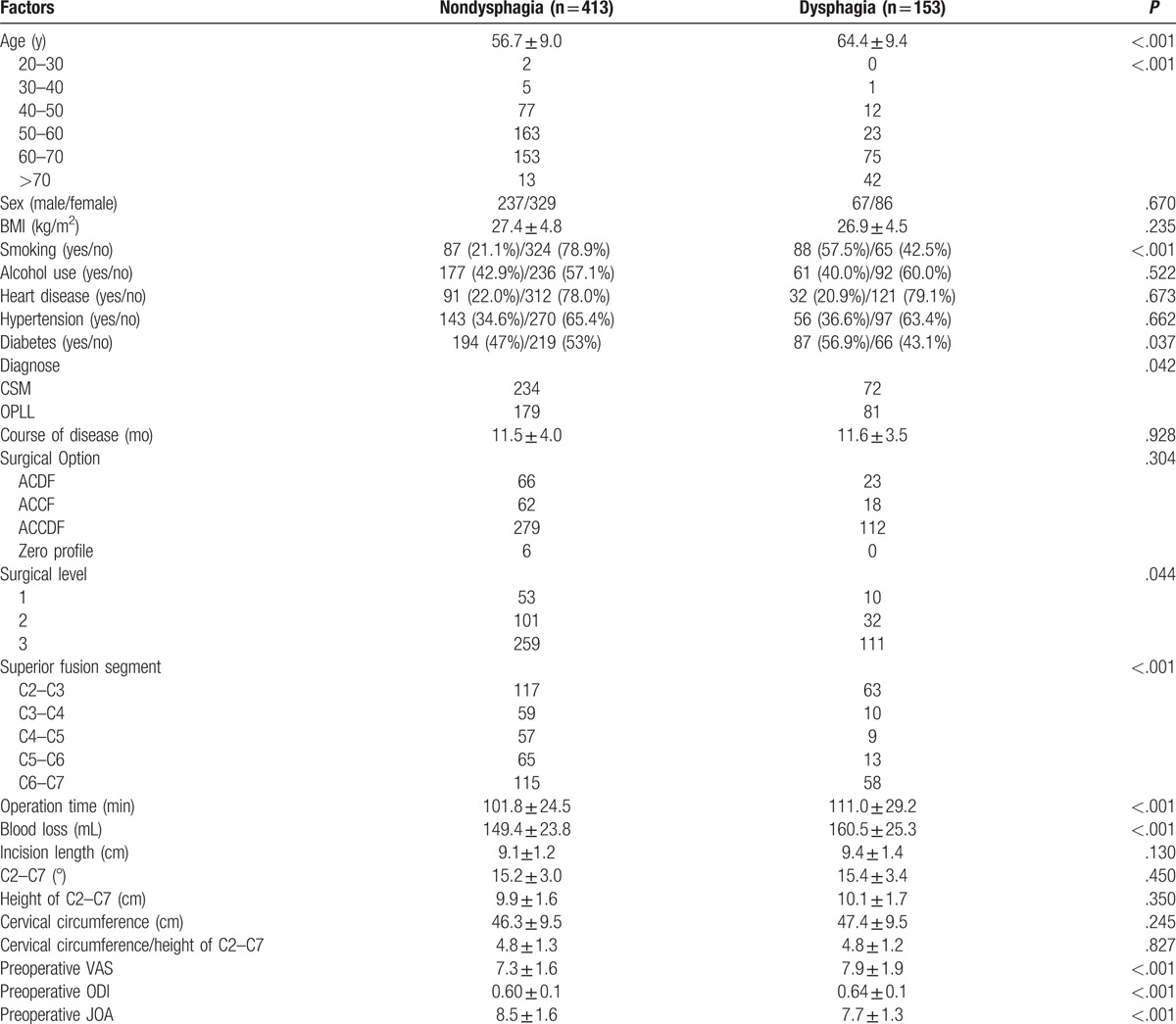
Comparison between nondysphagia group and dysphagia group 3 months after surgery.

The rate of dysphagia after anterior cervical surgery (6 months after surgery) was 2.4% (68 of 2827). Our results showed that age (67.6 ± 8.8), operation time (119.8 ± 25.7), blood loss (167.2 ± 24.0), preoperative VAS (8.5 ± 1.7), and ODI (0.66 ± 0.09) in dysphagia group were significantly higher than those (61.8 ± 9.0, 104.1 ± 30.0, 155.2 ± 25.2, 7.3 ± 1.8, 0.61 ± 0.10, respectively) in nondysphagia group; however, preoperative JOA (7.3 ± 1.2 vs 8.0 ± 1.3) had opposite trend between 2 groups. We could also infer that old subjects, individual with smoking, diabetes, and OPLL were the risk factors for dysphagia after 6 months of surgery, as shown in Table 3.

**Table 3:**
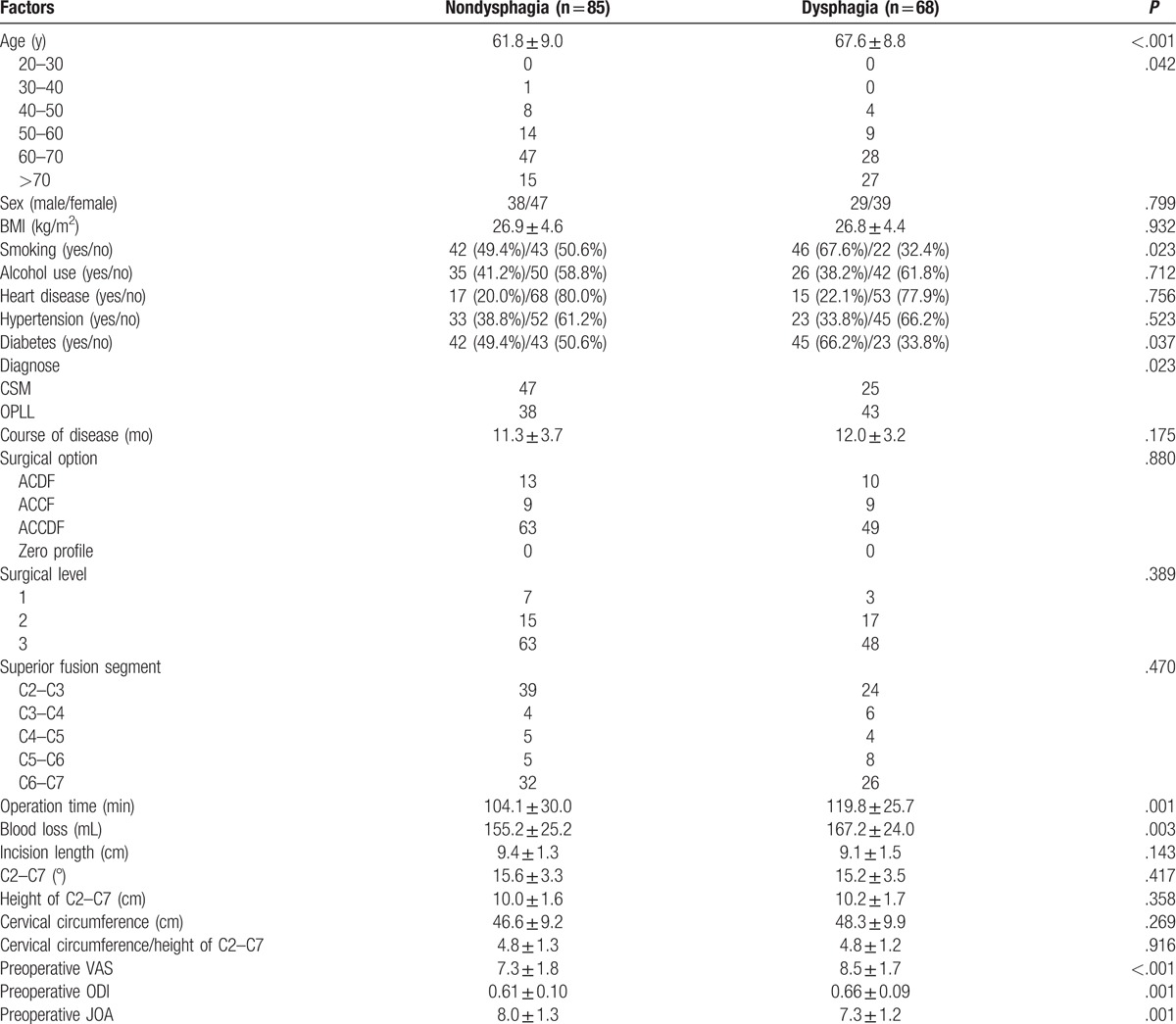
Comparison between nondysphagia group and dysphagia group 6 months after surgery.

The rate of dysphagia after anterior cervical surgery (1 year after surgery) was 1.1% (30 of 2827). Our results showed that age (70.0 ± 8.3), operation time (127.2 ± 21.2), and blood loss (176.3 ± 23.1) in dysphagia group were significantly higher than those (65.7 ± 8.9, 113.3 ± 27.4, 159.7 ± 22.1, respectively) in nondysphagia group. We could also infer that individual with smoking and diabetes were the risk factors for dysphagia 1 year after surgery, as shown in Table 4. The rate of dysphagia after anterior cervical surgery (2 years after surgery) was 0.4% (12 of 2827). Our results showed that smoking and diabetes in dysphagia group were significantly higher than those in nondysphagia group 2 years after surgery, as shown in Table 5.

**Table 4:**
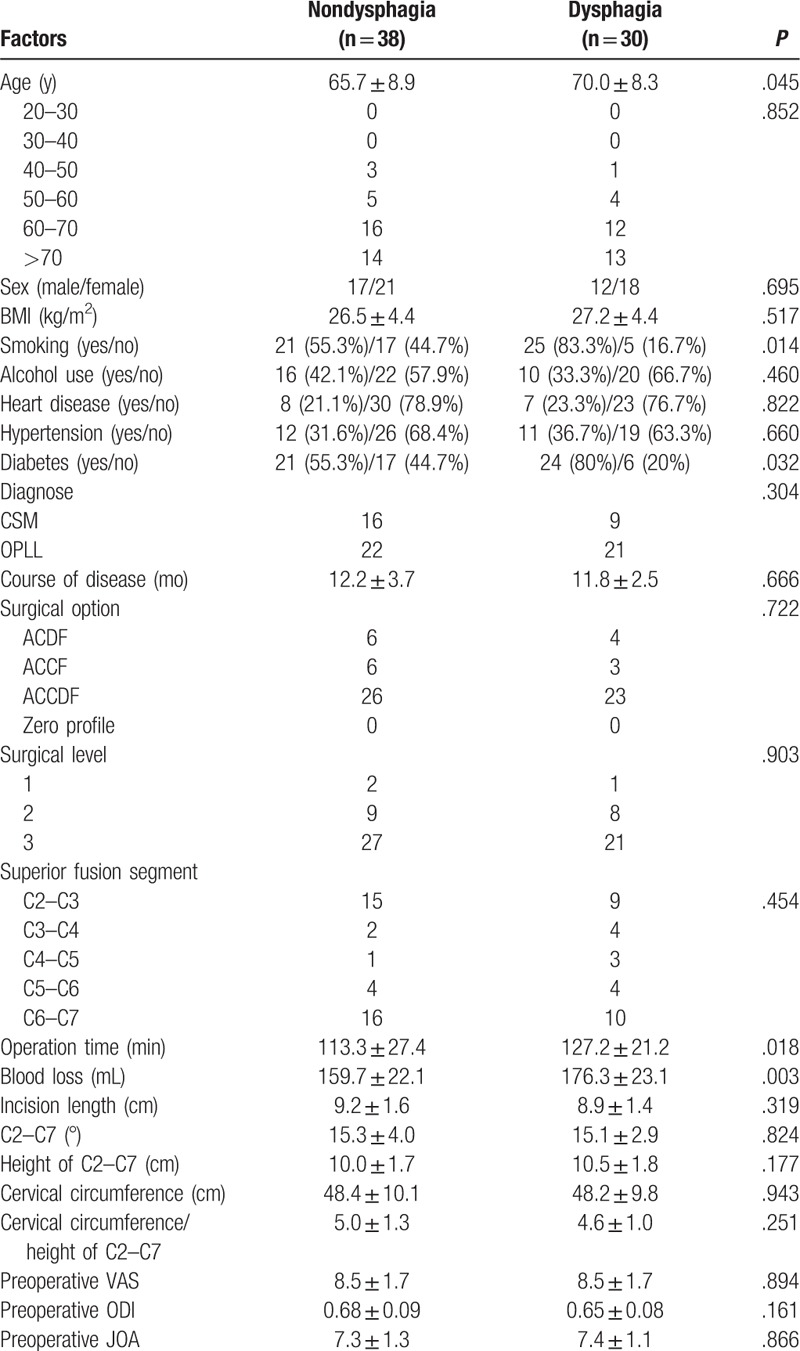
Comparison between nondysphagia group and dysphagia group 1 year after surgery.

**Table 5:**
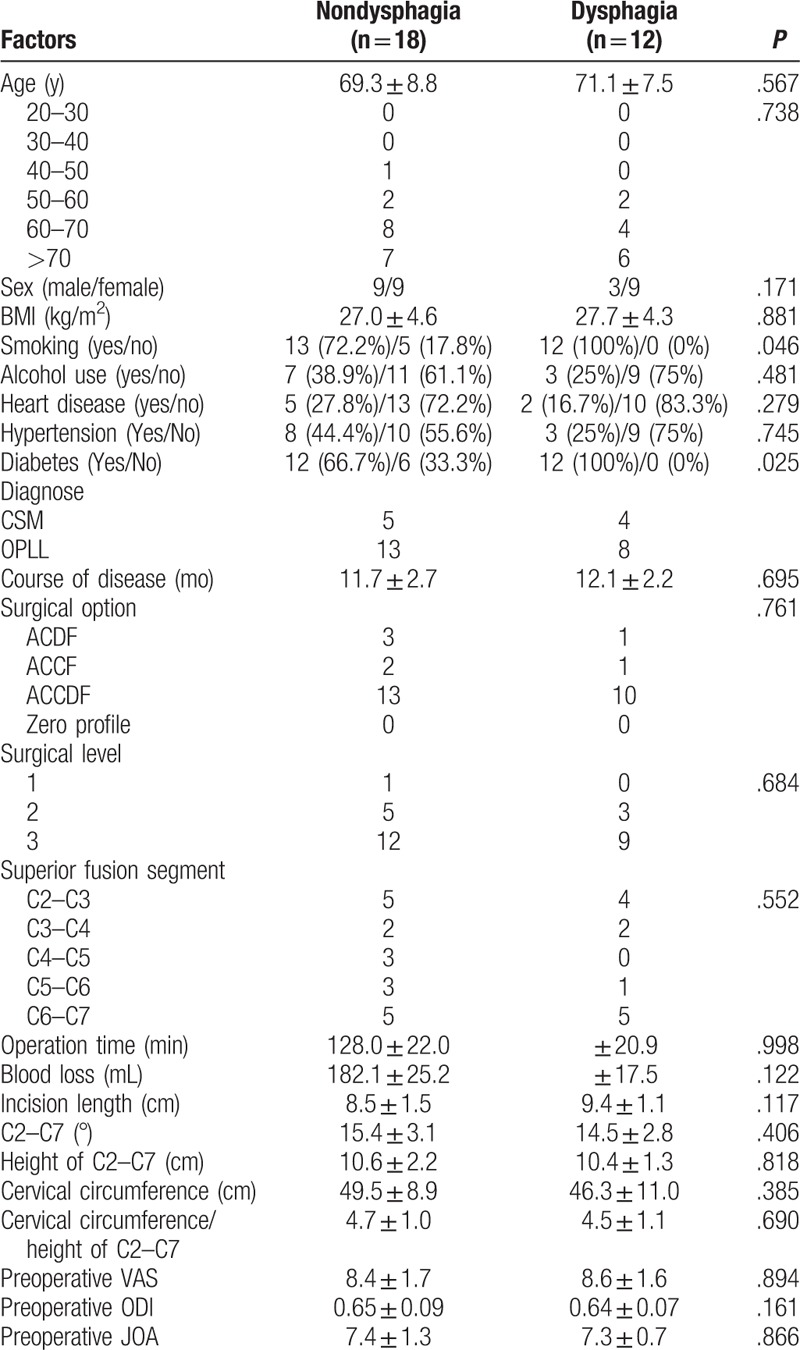
Comparison between nondysphagia group and dysphagia group 2 years after surgery.

## 4 Discussion

Dysphagia is a terrible complication after anterior cervical surgery, which can interfere daily and social life, and lower the satisfaction on surgery and quality of life if it continues for an extended period. We reviewed some related literature and concluded some points that may be the etiology of dysphagia, including pressure and long time of excessive retraction during operation for esophageal retraction, esophageal ischemia, irritation and inflammation caused by the height or the anterior profile of instrumentation. And other factors, like differences in the postoperative cervical kyphoticlordotic deformity and soft tissue edema and fibrosis due to postoperative granulation and fibrosis, also affect dysphagia after surgery.^[6–10]^ Postoperative dysphagia is caused by multiple factors, but the risk factors of it remain controversy.

Previous studies focused on single-center retrospective study, short follow-up, or small sample size. To our knowledge, this was the first multicenter retrospective study with large sample on this topic. The aim of our study was to explore risk factors of dysphagia after anterior cervical approaches and perioperative factors predicting on rehabilitation of dysphagia. The results of our study indicated that the rate of dysphagia at 0, 3, 6, 12, and 24 months after surgery were 20%, 5.4%, 2.4%, 1.1%, and 0.4%, respectively. Old subjects, female patients, patients with relatively higher BMI, smoking, diabetes, OPLL, longer course of disease, ACCDF, multilevel surgery, more operation time, more blood loss, longer incision length, more cervical circumference, larger angle of C2 to C7, higher preoperative VAS and ODI, lower JOA, shorter height of C2 to C7, and superior fusion segment including C2 to C3 or C6 to C7 were the risk factors for dysphagia after surgery immediately. However, 1 year after surgery, old people, more operation time and blood loss, and individual with smoking and diabetes were the risk factors. And 2 years after surgerysmoking and diabetes were only 2 risk factors for rehabilitation of dysphagia.

As for immediate postoperative dysphagia, blood loss, incision length, course of disease, patients with OPLL, ACCDF and multilevel surgery, preoperative VAS and ODI, JOA, BMI, and cervical circumference were risk factors. We considered operation time as the most one. Higher preoperative VAS and ODI, lower JOA, longer course of disease implied that state of an illness was relatively serious, which was easy to understand that we need more time to decompress and restore alignment. Similarly, for patients with higher BMI, more cervical circumference, more time to be need to thoroughly expose and peel soft tissue clearly. It is well known that patients with OPLL, ACCDF, superior fusion segment including C2 to C3 or C6 to C7 and multilevel surgery, which increase difficulty of surgery, we need more time to complete it perfectly. More operation time means excessive retraction, excessive duration and length of time of esophageal retraction, more serious esophageal ischemia, and easier to irritate soft tissue.^[10]^ More angle of C2 to C7 implied that anterior plates and instrumentation were more likely to irritate and surrounding soft tissue leading to inflammatory reaction. As the same with Bazaz,^[7]^ old patients and female patients were the risk factors for postoperative dysphagia.

Brad^[9]^ compared cervical arthroplasty and ACDF for dysphagia and found that no-profile cervical disc arthroplasty had a significantly lower rate of dysphagia. McAfee^[10]^ performed a prospective randomized on the same topic and draw the same conclusion. Xiao^[11]^ found that Zero profile could reduce the incidence of postoperative dysphagia compared with ACDF. We also observed 4 anterior surgical plans, including ACDF, ACCF, ACCDF, and Zero profile. Only 5 of 285 (1.7%) patients receiving Zero profile had postoperative dysphagia, which had markedly lower incidence of dysphagia than other options. Patients with ACCDF had the highest rate (51.7%, 387 of 749). We used to choose ACCDF for these patients with multilevel CSM, indicating that we spend more operation time accomplishing it. Excessive retraction and serious esophageal ischemia caused postoperative dysphagia, which also needed more time to recover. Besides, we found that patients with superior fusion segment including C2 to C3 or C6 to C7 had a higher rate than other segments, which was opposite to Samuel Kalb.^[12]^ We believed that compared with superior fusion segment including C3 to C4, C4 to C5, or C5 to C6, more retraction was needed to complete exposure for surgical vision in patients with superior fusion segment including C2 to C3 or C6 to C7.

Few articles considered smoking as an important factor for dysphagia after anterior cervical surgery. Olsson^[8]^ compared smokers with nonsmokers in dysphagia and showed that smokers were more likely to have dysphagia, and their dysphagia scores were more severe than those in nonsmokers. We surprisingly found that patients with smoking were always the risk factor for dysphagia at 0, 3, 6, 12, and 24 months. In addition, the rate of dysphagia in patients with smoking immediately after surgery was 27.4% (175 of 566), but the rate kept approximately 50% from 3 to 24 months. What is more, the proportion of smoking in patients with dysphagia was increasing from 0 (30.9%, 175 of 566) to 24 months (100%, 12 of 12). As mentioned earlier, the smoking was a vital risk factor for dysphagia and recovery for dysphagia. Our results is consistent with Olsson's.^[8]^

The present study has several limitations. First, it is a retrospective study; we need to conduct a prospective study to further explore the risk factors for dysphagia after anterior cervical surgery; second, some factors could not be observed due to retrospective study, for example, whether VAS, ODI, and JOA at time of 3, 6, 12, and 24 months after surgery could increase risk of SSI or not; whether reducing retraction properly during operation could lower risk of dysphagia or not, and so on. However, even though it has these limitations, it is valuable for surgeons to notice some variables leading to dysphagia before surgery.

We performed a multicenter retrospective study with large sample on dysphagia after anterior cervical surgery for 2 years of follow-up. The rate at 0, 3, 6, 12, and 24 months after surgery was 20%, 5.4%, 2.4%, 1.1%, and 0.4%, respectively. Many factors could increase risk of dysphagia after anterior cervical surgery, but we regarded operation time as the most important factor for immediate postoperative dysphagia and smoking as the most important factor for recovery of dysphagia. A prospective study is needed to assess factors for dysphagia after anterior cervical surgery.
